# Genomic and epigenetic evidence for oxytocin receptor deficiency in autism

**DOI:** 10.1186/1741-7015-7-62

**Published:** 2009-10-22

**Authors:** Simon G Gregory, Jessica J Connelly, Aaron J Towers, Jessica Johnson, Dhani Biscocho, Christina A Markunas, Carla Lintas, Ruth K Abramson, Harry H Wright, Peter Ellis, Cordelia F Langford, Gordon Worley, G Robert Delong, Susan K Murphy, Michael L Cuccaro, Antonello Persico, Margaret A Pericak-Vance

**Affiliations:** 1 Duke Center for Human Genetics, DUMC, Durham, NC, USA; 2 Laboratory of Molecular Psychiatry & Neurogenetics, University Campus Bio-Medico, Rome, Italy; 3 IRCCS 'Fondazione Santa Lucia', Rome, Italy; 4 Department of Neuropsychiatry, SOM-USC, Columbia, SC, USA; 5 Wellcome Trust Sanger Institute, Hinxton, UK; 6 Duke Department of Medicine, DUMC, Durham, NC, USA; 7 Departments of Obstetrics and Gynecology, and Pathology, Duke University, Durham, NC, USA; 8 John P Hussman Institute for Human Genomics, University of Miami Miller School of Medicine, Miami, FL, USA

## Abstract

**Background:**

Autism comprises a spectrum of behavioral and cognitive disturbances of childhood development and is known to be highly heritable. Although numerous approaches have been used to identify genes implicated in the development of autism, less than 10% of autism cases have been attributed to single gene disorders.

**Methods:**

We describe the use of high-resolution genome-wide tilepath microarrays and comparative genomic hybridization to identify copy number variants within 119 probands from multiplex autism families. We next carried out DNA methylation analysis by bisulfite sequencing in a proband and his family, expanding this analysis to methylation analysis of peripheral blood and temporal cortex DNA of autism cases and matched controls from independent datasets. We also assessed oxytocin receptor (OXTR) gene expression within the temporal cortex tissue by quantitative real-time polymerase chain reaction (PCR).

**Results:**

Our analysis revealed a genomic deletion containing the oxytocin receptor gene, *OXTR *(MIM accession no.: 167055), previously implicated in autism, was present in an autism proband and his mother who exhibits symptoms of obsessive-compulsive disorder. The proband's affected sibling did not harbor this deletion but instead may exhibit epigenetic misregulation of this gene through aberrant gene silencing by DNA methylation. Further DNA methylation analysis of the CpG island known to regulate *OXTR *expression identified several CpG dinucleotides that show independent statistically significant increases in the DNA methylation status in the peripheral blood cells and temporal cortex in independent datasets of individuals with autism as compared to control samples. Associated with the increase in methylation of these CpG dinucleotides is our finding that *OXTR *mRNA showed decreased expression in the temporal cortex tissue of autism cases matched for age and sex compared to controls.

**Conclusion:**

Together, these data provide further evidence for the role of OXTR and the oxytocin signaling pathway in the etiology of autism and, for the first time, implicate the epigenetic regulation of *OXTR *in the development of the disorder.

See the related commentary by Gurrieri and Neri:

## Background

Classic autism comprises a spectrum of behavioral and cognitive disturbances of childhood development. The core autism phenotype includes deficits in social interaction, language development and patterns of repetitive behaviors and/or restricted interests. The population prevalence of the spectrum of autism disorders is estimated to range between 1/300 [1] to 1/100 , with a male: female ratio of 4:1 [2,3]. The disorder has been shown to be highly heritable with the relative risk for siblings being approximately 2% to 8%, much higher than that of the general population [4]. To date, only a small percentage of autism cases (<10%) have been ascribed to single gene disorders such as fragile X syndrome, tuberous sclerosis [5] and Rett syndrome [6]. Numerous approaches including genetic linkage, genome-wide association, candidate gene association and gene expression analysis have been used to identify the additional genes implicated in the development of autism [7,8]. However, the heterogeneous nature of autism and autism spectrum disorders has limited their success.

An additional approach to identify genes involved in autism is to characterize copy number variants (CNVs), that is, chromosomal deletions and duplications, that are known to be present within at least 5% of individuals with idiopathic autism [9]. Autism CNVs have been shown to involve almost all chromosomes [10,11], with the most frequently observed alteration localizing to chromosome 15q11-13 [12-23]. A number of different methods have been used to characterize autism related CNVs, including but not limited to, cytogenetic G-banding [14,23,24], metaphase fluorescence *in situ *hybridization (FISH) [22], Southern blotting [18], loss of heterozygosity (LOH) analysis [15-17,19], quantitative polymerase chain reaction (PCR) [25] and, more recently, genotyping and representational oligonucleotide microarray analysis (ROMA) [26].

Here we describe the use of genome-wide tilepath microarrays and array comparative genomic hybridization (CGH) to identify CNVs in a dataset of 119 unrelated probands from multiplex autism families [27]. The genomic profiles of our autism dataset were compared to the array CGH profiles of 54 phenotypically normal individuals, to previously published CNVs present within the database of genomic variants [28] and to the Autism Chromosome Rearrangement Database . The most significant finding thus far from our analysis is a heterozygous deletion of the oxytocin receptor gene (*OXTR*) (MIM accession no.: 167055) in an individual with autism and his mother with putative obsessive-compulsive disorder (OCD). We further investigated the relationship between *OXTR *and autism by carrying out epigenetic analysis of the promoter region of *OXTR*. We show that the gene is hypermethylated in independent cohorts with autism as compared to controls, in both peripheral blood mononuclear cells (PBMCs) and the temporal cortex. Additionally, our analysis of expression levels of *OXTR *in the temporal cortex shows decreased levels of expression in individuals with autism as compared to controls matched for age and sex.

These data suggest that OXTR and the oxytocin signaling pathway play an important role in the etiology of autism and autism spectrum disorders and implicate epigenetic misregulation of *OXTR *in this complex disease.

## Methods

### Diagnostic and Statistical Manual of Mental Disorders criteria

Research diagnostic classification entailed the collection of detailed family, developmental, and medical history and systematic screening of each child with autism before proceeding with clinical assessments. The clinical diagnosis of autism was confirmed by standardized diagnostic assessments once a family had completed the screening process and was considered eligible. For research classification, a clinical diagnosis that conforms to Diagnostic and Statistical Manual of Mental Disorders, fourth edition (DSM-IV) criteria was made based on all available medical, clinical, and behavioral data. The clinical diagnosis was established as a research diagnosis on the basis of scores that exceed established cut-offs for autism on the Autism Diagnostic Interview-Revised (ADI-R) [29,30]. In addition, all individuals demonstrated a minimum IQ equivalent of 35 or Vineland Adaptive Behavior Scale age equivalent of 18 months. This ensured a sufficient developmental level for reliable classification on diagnostic measures. All instruments were administered by clinical staff that had been formally trained on all measures and, as appropriate, had demonstrated reliability within the research group as well as at clinical centers. All individuals are qualified only after review of materials by a licensed child psychologist (MC). Parents/caregivers were informed of the purposes, risks, and benefits of participating in this project and provided informed consent.

### Peripheral blood collection

All aspects of the research study were approved by the Institutional Review Boards of the participating centers. DNA for copy number and methylation analyses was obtained from the peripheral blood of autism probands from multiplex families, the extended family of the proband with the *OXTR *deletion, and phenotypically normal controls. Genomic DNA was extracted from whole blood using the Pure Gene method and standard protocols, as described previously [31]. DNA for the methylation analysis was collected from 20 autism cases and controls, all individuals were Caucasian, and the male to female ratio was 1:1.

### Autism brain tissue

Postmortem studies were performed using frozen brain tissue samples dissected from the superior temporal gyrus (BA 41/42) of eight patient-control pairs, obtained through the Autism Tissue Program from the Maryland National Institute of Child Health and Human Development (NICHD) Brain Tissue Center and the Harvard Brain Tissue Resource Center (Table 1). This neocortical region was chosen because it hosts well documented structural and functional abnormalities in autism [32-35]. These tissue samples largely overlap with those employed in our recent studies of the *MET *and *PRKCB1 *genes [36,37]. Clinical and demographic information, family history, and autopsy reports were obtained from the Autism Tissue Program web site  and are summarized in Table 1. The presence of mental retardation was defined on the basis of a full-scale IQ <70. Autism spectrum disorder cases fulfill DSM-IV diagnostic criteria [38], confirmed using the ADI-R [29,30]. Although the methylation study was not planned according to a matched design, all controls were selected to match patients on sex, age (± 2 years, wherever possible) and postmortem interval (PMI), where possible (Table 1).

**Table 1:** Description of clinical details associated with the temporal cortex tissue used in the methylation and expression analyses

**Case no**.	**Sex**	**Age (years)**	**PMI (h)**	**Diagnosis**	**Cause of death**
Controls:					
UMB-1185*	*M*	*4*	*17*	*Control*	*Drowning*
UMB-1860*	M	8	5	Control	Cardiac arrhythmia
B-3829*	M	22	24	Control	Central hepatic laceration
UMB-1706*	F	8	20	Control	Rejection of heart transplant
UMB-1377	F	5	20	Control	Drowning
B-6207	M	16	26	Control	Ischemic heart attack
B-6221	M	22	24	Control	Unknown
B-5873	M	28	23	Control	Unknown
B-4211	M	30	23	Control	Cardiac arrhythmia
Autism:					
B-5569*	*M*	*5*	*25.5*	*PDD-NOS*	*Drowning*
UMB-4721*	M	8	16	Autism	Drowning
B-5144*	M	20	23.7	Autism	Traffic accident
UMB-4671*	F	4	13	Autism	Accident
UMB-1174	F	7	14	Autism	Sudden death, seizure
B-6294	M	16	Unknown	Autism	Unknown
B-6337	M	22	25	Autism	Choking
B-5000	M	27	8.3	Autism	Drowning
B-5173	M	30	20	Autism	Gastrointestinal hemorrhage

Paired samples for expression analysis are denoted by an asterisk. Italicized samples were excluded from the methylation analysis to maintain phenotypic homogeneity.

PMI = postmortem interval; PDD-NOS = Pervasive Developmental Disorder Not Otherwise Specified.

### Array hybridization

High-resolution genome tilepath microarrays were constructed using large bacterial clones (bacterial artificial chromosome (BAC) clones) as previously described [39]. Briefly, BAC DNA was purified by alkaline lysis and used as the template for three different, degenerate oligonucleotide primed PCR reactions, prior to a second round of amino linking PCR and printing onto Codelink slides (GE Bioscience, Piscataway, NJ, USA) using a Genetix Qarray2 (Genetix, Boston, MA, USA). A total of 450 ng of autism proband and control DNA, from peripheral blood, were differentially labeled with Cy3-CTP and Cy5-CTP (BioPrime Labeling Kit; Invitrogen, Carlsbad, CA, USA), purified (Qiagen, Hilden, Germany) and hybridized to the tilepath arrays using the MAUI hybridization station (BioMicro Systems Inc, Salt Lake City, UT, USA). Image capture of the hybridized arrays for fluorescent intensity extraction was performed using a Genepix 4100A scanner (Molecular Devices, Sunnyvale, CA, USA) and Bluefuse microarray software (BlueGnome ) was used for data processing of the scanned images prior to porting into Nexus (BioDiscovery ) for analysis.

### Array CGH analysis

Microarray data was preprocessed in Nexus by removing poor quality flagged spots (confidence <0.3 as defined by Bluefuse software), removing background by Lowess correction before normalization of log_2 _ratios and combining BAC replicates. BioDiscovery's rank segmentation algorithm (RSA), which is similar to circular binary segmentation (CBS) [40], was used to identify genomic rearrangements. Briefly, the algorithm uses a normal distribution function for testing for change points as opposed to the non-parametric permutation based statistics used in the original CBS algorithm. It also uses the log ratio rank of each probe as oppose the log ratio value itself. The calling algorithm used cluster values and defined log_2 _thresholds of ± 1.4 and 0.38 for two and one copy gain/loss, respectively. We applied a conservative cut-off of three BAC clones showing the same copy number change trend to define genomic deletion or duplication with the significance threshold set at 0.005. Array CGH dye-swap experiments were carried out on 21 samples to confirm 21 genomic changes, including novel and known CNVs within the database of genomic variants.

### Quantitative real-time PCR validation

DNA copy number was validated using the Applied Biosystems 7900 HT Fast Real-Time PCR System (Applied Biosystems, Foster City, CA, USA). Primers and probes were designed to a region spanning exon 2 in *OXTR*: forward 5'-GCTGAACATCCCGAGGAACTG-3' and reverse 5'-GCAAATGAGCGGGAATCCTCTA-3'. DNA samples from autistic probands and their family members were used to determine if the CNV was familial or *de novo*. DNA isolated from tumor samples with known deletions at the regions containing *OXTR *were used as 1 × copy number controls and an unaffected individual identified in our normal-normal CGH experiment served as our 2 × copy number control at these loci. A primer and probe set designed to the single copy gene that encodes the RNA moiety for the RNase P enzyme, *RPPH1 *(MIM accession no.: 608513), a region of stable copy number (2 ×), was used to normalize each sample for equal input. Copy number was assessed in quadruplicate for each sample and the 2^-ΔΔC^_T _method was used for analysis [41].

### Microsatellite screening

Microsatellite markers were analyzed in the family carrying the deletion of *OXTR *to determine maternal or paternal contributions. Five markers on chromosome 3 (D3S1489, D3S18, D3S4539, D3S4163, D3S3691) were fluorescently labeled using PCR (primers indicated in Additional file 1) and size fractionated on an Applied Biosystems 3730 capillary sequencer (Applied Biosystems, Foster City, CA, USA). Then, 1 μl of the PCR product was diluted 1/70 and run on an agarose gel to confirm amplification, and 1 μl of the diluted PCR product was added to 19 μl of HiDye Formamide/LIZ 500 Standard solution and loaded on to the capillary sequencer and analyzed using standard software.

### Methylation analysis

DNA isolated from blood or brain samples was bisulfite converted using a methylSEQr Bisulfite Conversion Kit (Applied Biosystems, Foster City, CA, USA). Primers were designed to bisulfite-converted regions of CpG islands in the promoter region of the *OXTR *gene and in the third intron that had previously been shown to exhibit different patterns of methylation among individuals [42] (Additional file 2). PCR was performed on bisulfite treated DNA using the following reaction conditions, 1 cycle: 95°C for 3 min, 55 cycles: 95°C for 1 min followed by 56.8°C for 1 min followed by 72°C for 3 min, and a final extension at 72°C for 5 min. The reaction was run on an agarose gel and the bands were gel extracted (Qiagen, Valencia, CA, USA) and TOPO TA cloned (Invitrogen, Carlsbad, CA, USA). Single clones were isolated and plasmids purified (Qiagen, Valencia, CA, USA). Sequencing was performed using the M13 forward primer provided with the TOPO TA cloning kit. On average, 10 individual clones were sequenced per amplicon in order to calculate percentage methylation at each of the CpG sites (note: 8 clones were used in OXTR family analysis). Bisulfite conversion was verified for each clone by assessing the C to T conversion of non-CpG sites. The average methylation level (%) was determined for each individual and these values were used to generate an average percentage methylation for each group, autism cases and normal controls, respectively (Additional file 1). The Welch-Satterthwaite t test was employed to adjust for unequal variances between groups and to compare the mean methylation level (%) at each site between the autism cases versus control groups. A nominal significance of *P *≤ 0.05 indicated a significant change in the methylation state between the two groups. The direction of the change was determined by comparing the average methylation level at each site between the two groups.

### Expression analysis by quantitative PCR (qPCR)

Total RNA was extracted from four case-control pairs matched for age, sex and PMI (see Table 1) using the TRIzol reagent (Invitrogen, Carlsbad, CA, USA) according to standard methods, and RNA quality was checked using a Bioanalyzer (Agilent, Santa Clara, CA, USA). Reverse transcription was performed with the QuantiTect Reverse Transcription kit (Qiagen, Valencia, CA, USA), using random hexamers as primers and a starting RNA quantity of 1 μg in a 20 μl final volume. *OXTR *cDNA was quantified using an iQ5 Multicolour Real-time PCR Apparatus (Bio-Rad, Hercules, CA, USA), according to a standard ΔΔCt Syber Green protocol [43]. *PPIA *(MIM accession no.: 123840) cDNA was measured in parallel and used as a standard for normalization. Due to relatively low neocortical *OXTR *gene expression levels compared to *PPIA*, PCR reactions were performed using 1:5 and 1:25 cDNA dilutions, respectively. *OXTR *primers were targeted to exon 2, which is shared by all four alternative mRNA transcripts, and designed as follows: F2, 5'-CTGAACATCCCGAGGAACTG-3', and R2, 5'-CTCTGAGCCACTGCAAATGA-3'. To ensure specificity, PCR products were initially sequenced using a CEQ8000 DNA sequencer (Beckman-Coulter, Fullerton, CA, USA), and afterwards checked by running a melting curve at the end of each experiment. Analysis was performed using the 2^-ΔΔC^_T _method [41] and expression level from the group of cases and the group of controls were compared using a one-tailed paired t test.

## Results

### Normal-normal analysis

Array CGH data was generated for 54 phenotypically normal individuals in 27 sex-mismatched hybridizations to establish the performance of our large insert bacterial clones on the genomic tilepath microarray. Sex mismatching was used to establish the baseline of heterozygous loss using the X chromosome as a reference. Analysis of the 27 normal-normal array CGH experiments identified 12 CNVs within 2 or more of the array CGH hybridizations. In all, 10 CNVs were contained within previously published CNVs from the database of genomic variants [28], while 2 CNVs extended the size of the known CNV regions (Additional file 3).

### Autism CNVs

**Figure 1:**
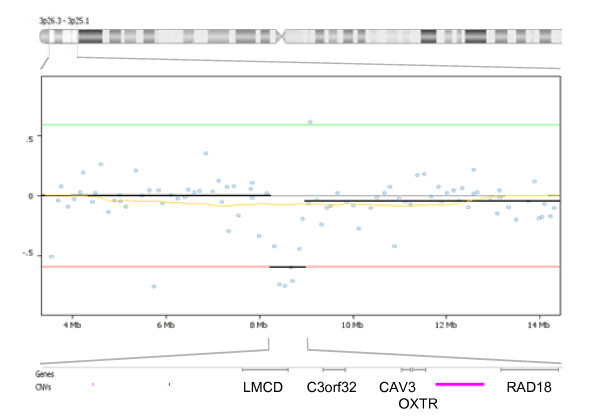
**Graphical representation of the 0.7 Mb heterozygous deletion in 3p25.3 (8,231,927-8,985,513 base pairs) within an individual with autism identified by array comparative genomic hybridization (CGH)**. The horizontal ideogram represents the chromosome 3 region of interest with the log_2 _plot below it of array CGH results from the tilepath clones (individual blue circles) on the genomic array. The dotted line at '0' represents copy neutral log_2 _plot between the cohybridized samples, while the green line represents single copy gain and the red line single copy loss. *OXTR *and four adjacent genes are contained within a deletion called in Nexus by circular binary segmentation (CBS) [40] (bold black line). Known copy number variants from the Database of Genomic Variants  are denoted by horizontal pink bars.

The genomic profiles of 119 idiopathic autism probands (93 male and 26 female) were generated by sex-mismatch array CGH experiments using controls from our normal-normal analysis. A total of 113 loci within 111 autistic individuals contained at least 1 genomic deletion or duplication, with a range of 1-6 CNVs per individual (Additional file 3). Approximately half of these, 52, were wholly contained within known copy number variants from the Database of Genomic Variants [28], 57 loci overlapped with 1 or more known CNVs regardless of size (CNV regions), and 5 were novel regions of loss (n = 2) or gain (n = 3) that contained 21 genes (Additional file 3). According to Fable  only one gene within these novel regions, ring finger protein 19A (RNF19A, also known as p38) in a 0.6 Mb deletion on chromosome 8q22.2, has been previously implicated in autism spectrum disorders (ASDs) via an observation in Rett syndrome [44], and none fell within previous regions of linkage (as reviewed in [7]). We chose to focus on 19 of the 113 putative deletions or duplications identified in the first screen to validate by array CGH dye swap experiments using select individuals (Additional file 3, top) or, in the case of the *OXTR *deletion described below, by microsatellite and quantitative real-time PCR analysis. Genomic rearrangements of note included a 12.5 Mb deletion within 2q24.1-2q24.3, a region previously linked to autism [45,46] and containing 41 known genes; a 4.6 Mb duplication and a 3.4 Mb deletion within 15q11-13 that contains a complex arrangement of CNVs and has previously been associated with autism by numerous genetic linkage [47-49] and genome copy number studies [12-23]; and a 0.7 Mb deletion in 3p25.3 that contains 5 genes: Lim and cysteine-rich domains 1, *LMCD1 *(MIM accession no.: 604859); C3orf32; Caveolin 3, *CAV3 *(MIM accession no.: 601253); oxytocin receptor, *OXTR *(MIM accession no.: 167055); and *RAD18 *(MIM accession no.: 605256) (Figure 1). Though this region contains known smaller copy number variants, none of these correspond to a deletion of 0.7 Mb identified by this study or contain the genes mentioned above. Additionally, *OXTR *is a candidate autism gene whose levels may be important for social development of offspring [50].

### Copy number analysis of *OXTR*

**Figure 2:**
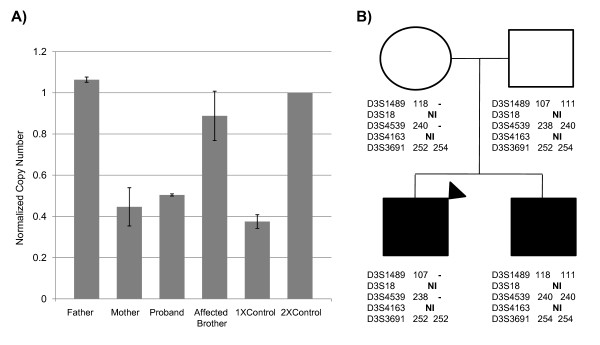
**Microsatellite and quantitative real-time polymerase chain reaction (PCR) validation of *OXTR *deletion**. **(a) **Microsatellite markers within and adjacent to the copy variable region in the *OXTR *deletion family were assayed to confirm homozygosity and parental transfer of alleles. **(b) **Normalized real-time PCR results confirm the loss of a single copy of *OXTR *in both the mother and the proband (marked by a black triangle). Copy number was normalized to RNAseP and controls were used to verify deletion. The 1 × control is a tumor sample with a known chromosome 3 deletion. The 2 × control is an unaffected sample from the copy number variant (CNV) study in this paper that is not copy variable at *OXTR*.

Based on the strength of the novel deletion of *OXTR *and the previous data implicating OXTR in the development of autism [51-53], we focused further copy number analysis on the 3p25.3 deletion by screening other members of the proband's family. Quantitative real-time PCR (qRT-PCR) analysis confirmed that the deletion containing *OXTR *was not a *de novo *event (Figure 2a) and microsatellite analysis confirmed that the deletion was inherited from the mother (Figure 2b), who has possible OCD (not clinically evaluated). OCD cases have been reported to be more common among first-degree relatives of autistic probands, which may hint at a common genetic mechanism or pathway shared between these two disorders [54,55]. Curiously, the proband's affected brother did not inherit the allele containing the genomic deletion (Figure 2). Microsatellite analysis of the deletion revealed that the proband's alternative allele was inherited from the father, therefore excluding the prospect of uniparental disomy at the *OXTR *locus contributing to the phenotype (Figure 2).

### Methylation analysis of the *OXTR *promoter region

**Figure 3:**
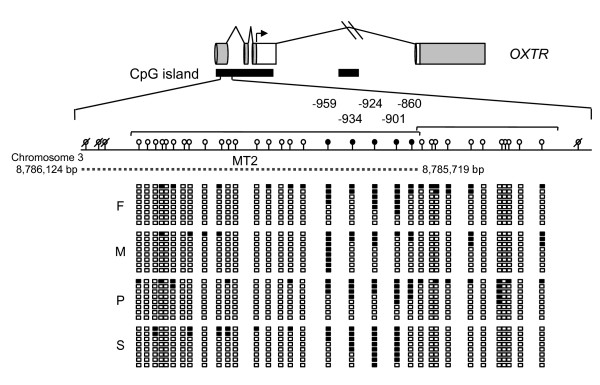
**CpG dinucleotides analyzed within a predicted CpG island in the promoter region of *OXTR***. Untranslated regions are represented as gray boxes and protein coding exons are white boxes, the site of translation is denoted by a black arrow. Predicted CpG islands are horizontal black bars beneath the gene. The region of interest within the 5' CpG island, MT2 as reported by Kusui *et al*. [56], is denoted by a dashed line (NCBI36 coordinates). Groups of CpGs sequenced per clone are indicated with brackets. Clear circles with diagonal lines denote untested CpG dinucleotides, filled circles represent region of interest. CpGs of interest are numbered according to translation initiation site of +1. Clear boxes denote CpG dinucleotides that showed no methylation in bisulfite sequencing of subclones; black within boxes denotes methylation of the CpG dinucleotide; F = Father, M = Mother, P = Proband, S = Sibling.

Based on the CNV data within the *OXTR *deletion family, we hypothesized that a hemizygous deletion of *OXTR *may result in a reduction in the available levels of OXTR *in utero *and during development, ultimately leading to the development of autism. However, if this is true, it is unclear how this deletion confers autism in the proband when the proband's affected sibling does not carry the deletion. To investigate the possibility that a mechanism other than a genomic deletion may play a role in decreasing *OXTR *levels in the affected sibling we investigated the DNA methylation status, a form of epigenetic silencing, of two regions of *OXTR *previously shown to be methylated and associated with differential expression of the gene in liver and myometrium [56,57]. We carried out bisulfite sequencing (BSS) analysis of cloned alleles of two *OXTR *CpG islands in the peripheral blood mononuclear cells (PBMCs) of all four family members (Figure 3). The first CpG island overlaps exons 1, 2, and 3 of *OXTR *and is located on chromosome 3 at 8,783,962-8,786,280 base pairs (Ensembl Homo sapiens version 54.36p, NCBI36). Kusui *et al*. had shown that the methylation status of a specific 405 base-pair fragment of this CpG island (termed 'MT2' by Kusui *et al*.) is important for tissue-specific expression of *OXTR *(Figure 3 depicts MT2 position). The second CpG island is located within the third intron of *OXTR *on chromosome 3 at 8,775,160-8,776,624 base pairs (Ensembl Homo sapiens version 54.36p, NCBI36). This island has previously been experimentally defined as an *Eco*RI-*Kpn*I fragment that was less methylated in myometrial DNA than in the DNA from peripheral blood leukocytes [42]. All CpG dinucleotides tested within the intron 3 CpG island were heavily methylated in all family members (data not shown). However, several of the 33 CpG dinucleotides tested within the region overlapping exons 1, 2, and 3 of *OXTR *differed in methylation level between family members. Specifically, CpG dinucleotides at positions -959, -934, -924, -901 and -860 (relative to translation start site) showed the most dramatic differences in methylation status, with the proband's affected sibling being heavily methylated at sites -934, -924 and -901. These specific residues fall within the critical region (MT2) in the CpG island of *OXTR *shown by Kusui *et al*. to regulate the expression of *OXTR *in liver [56]. Importantly, in keeping with the hypothesis that increases in DNA methylation (hypermethylation) lead to gene silencing, CpG sites -934 and -924 showed the largest increase in methylation, 37.5%, between the autistic sibling and the non-autistic father (62.5% methylated versus 25% methylated, 100% methylated versus 62.5% methylated, respectively), both lacking the *OXTR *deletion. The 100% methylation of a specific CpG could indicate maintenance of an aberrant methylation mark, making this a more dramatic change.

### Hypermethylation of residues critical for *OXTR *gene silencing are associated with autism

Given the data observed within the *OXTR *deletion family, we hypothesized that DNA methylation of residues found within the critical region of *OXTR *regulation may extend beyond this family and represent a more general epigenetic contribution to autism spectrum disorders. We extended our BSS analysis of the 5 differentiallly methylated CpG dinucleotides to PBMCs from 20 phenotypically normal control individuals (10 males and 10 females) and 20 individuals with autism (10 males and 10 females). BSS data showed significantly increased methylation in the PBMCs of CpG -860 (*P *= 0.0009), -934 (*P *= 0.0081) and -959 (*P *= 0.0026) in individuals with autism compared to controls (Table 2). When the data are stratified by sex, the associated significance of -860 and -934 can be ascribed only to males (Table 3) and that of -959 to females (Additional file 4).

**Table 2:** *OXTR *promoter CpG methylation levels in peripheral blood mononuclear cells

**Site**	**AutD cases (95% CI), (n = 20)**	**Controls (95% CI), (n = 20)**	**t Test**
-860	35.1% (22.6 to 46.7)	12.2% (6.5 to 17.9)	**0.0009**
-901	52.0% (42.4 to 61.6)	45.2% (32.5 to 57.8)	0.3761
-924	68.1% (58.2 to 78.0)	65.8% (54.4 to 77.3)	0.7558
-934	56.4% (43.4 to 69.4)	33.4% (22.2 to 44.6)	**0.0081**
-959	50.9% (42.4 to 59.4)	32.0% (23.2 to 40.8)	**0.0026**

Bold text indicates a significant result by Satterthwaite test; % = percentage methylated.

CI = confidence interval; AutD = autism disorder.

**Table 3:** *OXTR *promoter CpG methylation status in peripheral blood mononuclear cells (PBMCs) and cortex samples of males

**Site**	**AutD cases (95%CI), (n = 10)**	**Controls (95%CI), (n = 10)**	**t Test**
PBMCs:			
-860	41.0% (28.4 to 53.7)	13.8% (6.6 to 21.0)	**0.0008**
-901	48.1% (33.6 to 62.7)	36.9% (19.1 to 54.7)	0.3153
-924	71.1% (59.4 to 82.8)	59.3% (40.8 to 77.8)	0.2604
-934	58.7% (39.4 to 78.1)	19.8% (3.2 to 36.4)	**0.0029**
-959	48.6% (39.5 to 57.6)	41.7% (27.5 to 55.9)	0.3522
Cortex:			
-860	38.8% (5.4 to 72.2)	14.9% (3.6 to 26.2)	0.1208
-901	47.1% (13.1 to 81.2)	13.3% (3.6 to 23.0)	0.0508
-924	64.3% (30.6 to 98.0)	26.6% (7.2 to 46.1)	**0.0375**
-934	61.0% (24.4 to 97.5)	19.4% (1.1 to 37.7)	**0.0320**
-959	20.5% (8.0 to 32.9)	19.4% (1.1 to 37.7)	0.9032

Bold text indicates a significant result by Satterthwaite test; %, percentage methylated. Cases and controls were all male.

CI = confidence interval; AutD = autism disorder.

### Hypermethylation of *OXTR *CpG sites in temporal cortex tissue of autistic individuals

To determine if differential methylation of the five putative regulatory *OXTR *CpG dinucleotides may be important in a tissue implicated in the etiology of autism, we analyzed temporal cortex tissue from eight individuals with autism and eight controls matched for age and sex (see clinical data in Table 1), six male and two female. The BSS results in the cortex again showed statistically significant hypermethylation in the autism cases versus normal controls. There was significant increased methylation at CpG -860 (*P *= 0.0251), CpG -901 (*P *= 0.0149), CpG -924 (*P *= 0.0448) and CpG -934 (*P *= 0.0233) between autism cortex tissue compared to the normal cortex controls (Additional file 5). We performed a stratified analysis to increase the homogeneity within the sample using six male samples matched for age and sex, and to validate our sex-specific findings in blood. This analysis reproduced the significance at sites -924 and -934 (Table 3).

### Expression analysis of *OXTR *in temporal cortex tissue of OXTR

**Figure 4:**
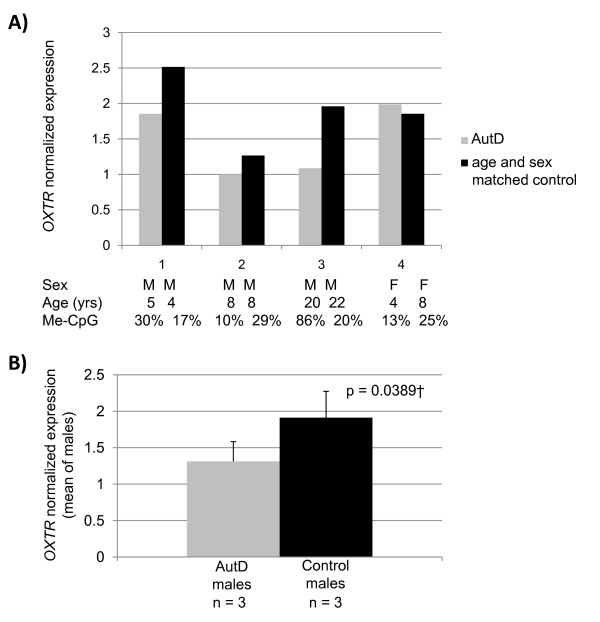
**Expression of *OXTR *in the cortex of male autistic brains is decreased compared to controls**. *OXTR *expression in the cortex was quantitated using quantitative real-time polymerase chain reaction (qRT-PCR) and normalized to *PPIA *in four cases (autism) and controls matched for age and sex. **(a) **The expression level of *OXTR *is decreased in male cases compared to controls and the methylation of site -934 (Me-CpG) correlates with expression in two of the male individuals. **(b) **As a group, the expression of *OXTR *in the cortex of the male autistic brain is significantly lower than in controls matched for age and sex. †Paired t test.

To functionally correlate DNA methylation levels within the temporal cortex DNA and expression of *OXTR *from which the DNA was derived, we carried out quantitative PCR analysis of mRNA levels from the cortex tissue of four autism cases matched for age and sex and four controls. Assays were designed to exon 2 of the gene. Although *OXTR *expression levels were low, we detected a sex-specific decrease in expression of *OXTR *for each of the autistic male control pairs tested (Figure 4a), and in two of the three cases expression levels correlated with an increase in methylation at site -934. As a group, autistic males showed a 20% decrease in expression compared to controls that was significant at *P *= 0.0389 (Figure 4b). These data suggest that increased methylation of the promoter in *OXTR *correlates with decreased expression of the gene in a tissue type relevant to the development of autism.

## Discussion

For several years the characterization of CNVs in autism patients has identified genes that potentially contribute to the etiology of the disorder [9,58-60]. In this study we used high-resolution genomic tilepath arrays with comparative genomic hybridization to identify 113 CNVs within 119 unrelated autistic individuals. Of note were five novel CNVs that do not map to prior regions of linkage and which, according to Fable , contain only one gene in 8q22.2 (RNF19A) that has previously been implicated in autism spectrum disorders [44]. CNVs observed in known autism regions included a 12.5 Mb deletion within 2q24.1-2q24.3 [45,46], two rearrangements in 15q11-13 [12-23,47-49], and a 0.7 Mb deletion in 3p25.3. We chose to focus on the deletion in chromosome 3p25.3 because of previous reported linkage to the region [61,62], association data implicating the oxytocin receptor (*OXTR*) that is contained within the deletion to the etiology of autism [51-53]. More recently, the DECIPHER database  has had copy number data deposited from a syndromic patient who has developmental delay and cognitive impairment presumably emanating from a novel 1.1 Mb deletion that contains *OXTR*, amongst other genes. Detailed DNA methylation analysis of the promoter region of *OXTR*, prompted by the observation that the proband's autistic brother did not carry the novel deletion, identified a single CpG dinucleotide within a known OXTR control region [56] that, independently, showed a statistically significant increase in methylation in PBMCs and the temporal cortex tissue of autism cases compared to controls. The identification of similar methylation profiles in the temporal cortex and peripheral blood may be indicative of an early developmental event during which these profiles are differentially established, prior to germ layer specification. If this locus is epigenetically labile, then it might be vulnerable to (unknown) exposures *in utero *during the first few weeks of pregnancy that lead to shifts in methylation status of OXTR. Similar influences have been seen in IGF2 methylation based on *in utero *exposures such as smoking and folic acid intake in humans [63]. Since epigenetic profiles undergo reprogramming during very early gestation (fertilization to implantation), it is not surprising that the profiles in these two disparate tissue types of ectodermal and mesodermal origin are similar and furthermore provides a potential means for detection in multiple, more accessible tissues.

The impact of this epigenetic mark on *OXTR *expression was supported by analysis of the corresponding mRNA from the temporal cortex of autism cases matched for age and sex and controls. As one would hypothesize given their increased methylation of *OXTR*, the autism cases also showed statistically lower transcript levels of *OXTR *expression compared to the normal controls. Because deletions encompassing *OXTR *have not been observed in other studies characterizing structural variation in autism [58,64,65] such events appear to be rare. At the same time our findings suggest that the hypermethylated status of *OXTR *in autism, resulting in a decrease in *OXTR *expression, may more commonly contribute to the disorder. While the mechanism for the development of autism from a germ line deletion of *OXTR *versus epigenetic silencing of the gene in the temporal cortex may clearly be different, the effect on oxytocin signaling could be the same. Together, these data provide strong evidence for the role of *OXTR *and oxytocin signaling pathway in the etiology of autism and, for the first time, show that the epigenetic regulation of *OXTR *may be an important factor in the development of the disorder.

Epigenetic control of autism susceptibility is a recent concept and most certainly a topic of great interest in the field. Over the past decade, researchers have uncovered suggestive links between epigenetics and autism, for example, autism is associated with duplications of 15q11-13 (especially maternally inherited), an imprinted region in the genome where DNA methylation status has been linked to Prader-Willi syndrome (PWS) and Angelman syndrome (AS) [66]; mutation within a gene that encodes a methyl-DNA-binding protein (*MECP2*, (MIM accession no.: 300005)) is the causative agent of Rett syndrome [67]; and mutation of this same gene has been associated with both autism and AS populations [55]. Nagarajan *et al *have shown that 79% of autism cases have a decrease in *MECP2 *expression in the frontal cortex and that an increase in aberrant DNA methylation correlates with this decrease in *MECP2 *expression [68]. These data implicate epigenetic dysregulation as a mechanism for the development of autism and justify the examination of DNA methylation of autism candidate genes, such as *OXTR *identified in this study.

The oxytocin receptor, *OXTR*, is spatially and temporally regulated by a complex combination of sex hormones, inflammatory cytokines, oxytocin (OXT) feedback and epigenetic mechanisms [56,57]. It is a high affinity G-protein coupled receptor for OXT, a nine amino acid hormone that has important and complex roles in animal physiology and behavior [69], including parturition and neurotransmission. A putative role for OXT in the development of ASDs was first highlighted by Insel and colleagues [70] who showed that signaling of oxytocin and the closely related molecule vasopressin play important 'prosocial' roles influencing social behavior and cognitive function in a species-specific manner [71-73]. Subsequent studies in mice revealed that *Oxt *[74] and *Oxtr *[75] knockouts largely resulted in normal parturition, however, social memory was impaired. Recently, Lee and colleagues [76] showed that only male mice with a targeted forebrain knock out of *Oxtr *exhibited social impairment by failure to recognize individuals of their own species. It has been suggested that the normal social behavior of the female knockout mice may be attributed to the compensatory function of vasopressin or developmental compensation [69,77]. In an excellent review by Carter [50] that describes the complex sexually dimorphic roles of vasopressin and oxytocin in brain function and behavior, it is apparent that aberrant oxytocin and/or vasopressin signaling is likely to elicit sex-specific outcomes, which is of particular relevance to autism because of disproportionate number of affected males. Our family analysis identified an *OXTR *deletion in the mother who does not present with autism. Sex-specific effects of OXTR function from animal models, together with statistical data that suggest dominantly acting *de novo *mutations may have reduced penetrance in females [78], may explain why the heterozygous deletion of *OXTR *in the mother from our study does not result in autism while the male proband displays the disorder.

We identified a single CpG dinucleotide within a predicted CpG island that showed statistically significant hypermethylation in PBMCs and the temporal cortex of individuals with autism compared to controls. Although the dominant paradigm for regulation of transcription by DNA methylation suggests that clusters of CpGs are necessary for silencing, recent evidence has indicated that methylation of single CpG dinucleotides may also lead to transcriptional changes via interference of transcription factor binding or recruitment of silencing factors [79]. This is the most likely explanation for the findings presented here as CpG site -934 falls within a predicted c-Rel, ZHX2, and LGALS4 binding domains (T. Wang, personal communication). However, further molecular work is required to dissect the likely complex transcriptional regulation of *OXTR*.

In this study, we focused on the temporal cortex (Brodmann areas 41/42 and 22) because studies have shown that localized temporal dysfunction is linked with clinical symptoms in autism, such as perceptive, emotional and cognitive deficits [32-35]. Although we were not able to determine the methylation status of cortex tissue from the family in which we observed the *OXTR *deletion, the data we collected from the affected sibling shows hypermethylation of the CpG dinucleotide (-934) in the predicted CpG island upstream of *OXTR*. Given our results suggesting that DNA methylation of *OXTR *in PBMCs may be a marker of methylation status in the temporal cortex, it follows that the affected sibling may also exhibit a decrease in *OXTR *expression due to methylation instead of deletion; thus implicating two separate mechanisms of transcriptional regulation with the same outcome. These results suggest that because the methylation status of site -934 is significantly different in the temporal cortex and PBMCs between autism cases and controls, measuring the methylation status of *OXTR *from the blood for autism cases could act as a surrogate for methylation status in the temporal cortex and be diagnostic of affection status, together with traditional diagnostic criteria.

## Conclusion

In summary, the data presented suggest that genomic deletion or aberrant methylation of *OXTR *may contribute to the development of autism. We acknowledge that screening for these genomic changes in a large, well controlled autism sample is necessary to determine the prevalence of these changes and their relationship to the disease. However, regardless of the sample size, we provide strong evidence that the epigenetic regulation of *OXTR *may be an important factor in the development of the disorder and suggest that further studies begin to focus on this regulatory event and its relationship to disease.

## Competing interests

The authors declare that they have no competing interests.

## Authors' contributions

SGG conceived the experimental design and wrote the paper; JJC assisted with experimental design, carried out experiments and wrote the paper; AT, JJ, DB, DB, CM and CL carried out the experiments; RKA, HHW, GW ascertained autism families for the paper; PE and CFL assisted with experimental design, SKM assisted with the writing of the paper; MLC helped ascertain autism families and assisted with clinical assessment of the study population; AP and MAP-V assisted with experimental design and in the writing of the paper.

## Pre-publication history

The pre-publication history for this paper can be accessed here:



## Supplementary Material

Additional file 1 **Cloned bisulfite sequencing results for each peripheral blood mononuclear cell (PBMC) sample.** Click here for file

Additional file 2 **Primer pairs used for this study.** Click here for file

Additional file 3 **Genomic duplication and deletion events within 54 control and 119 autistic individuals identified by whole genome tilepath array comparative genomic hybridization (CGH).** Click here for file

Additional file 4 **OXTR promoter CpG methylation status in peripheral blood mononuclear cells (PBMCs) of 10 autistic females and 10 age-matched controls.** Click here for file

Additional file 5 **OXTR promoter CpG methylation status in the cortex of eight autism cases and controls matched for age and sex.** Click here for file
